# Differential molecular biomarker expression in corals over a gradient of water quality stressors in Maunalua Bay, Hawaii

**DOI:** 10.3389/fphys.2024.1346045

**Published:** 2024-02-21

**Authors:** Kaho H. Tisthammer, Jonathan A. Martinez, Craig A. Downs, Robert H. Richmond

**Affiliations:** ^1^ Kewalo Marine Laboratory, University of Hawaii at Manoa, Honolulu, HI, United States; ^2^ U.S. Fish and Wildlife Service, Albuquerque, NM, United States; ^3^ Haereticus Environmental Laboratory, Clifford, VA, United States

**Keywords:** corals, coral reefs, biomarkers, protein expression, stress responses, environmental gradients, water quality

## Abstract

Coral reefs globally face unprecedented challenges from anthropogenic stressors, necessitating innovative approaches for effective assessment and management. Molecular biomarkers, particularly those related to protein expressions, provide a promising avenue for diagnosing coral health at the cellular level. This study employed enzyme-linked immunosorbent assays to evaluate stress responses in the coral *Porites lobata* along an environmental gradient in Maunalua Bay, Hawaii. The results revealed distinct protein expression patterns correlating with anthropogenic stressor levels across the bay. Some proteins, such as ubiquitin and Hsp70, emerged as sensitive biomarkers, displaying a linear decrease in response along the environmental gradient, emphasizing their potential as indicators of stress. Our findings highlighted the feasibility of using protein biomarkers for real-time assessment of coral health and the identification of stressors. The identified biomarkers can aid in establishing stress thresholds and evaluating the efficacy of management interventions. Additionally, we assessed sediment and water quality from the inshore areas in the bay and identified organic contaminants, including polycyclic aromatic hydrocarbons and pesticides, in bay sediments and waters.

## 1 Introduction

Coral reefs worldwide have been severely impacted by anthropogenic activities, ranging from global level stressors of climate change to various local stressors, including land-based sources of pollution resulting in reduced water quality, overfishing, and disease outbreaks (Wilkinson, 2004; Richmond and Wolanski, 2011; Hoegh-Guldberg, 2014; Birkeland, 2015). For coral reefs in Hawaii, reduced water quality is one of the most important local threats. As the health of coral reefs continues to deteriorate, we are facing the pressing need for effective and informative assessment tools for both diagnosing the effects of specific stressors and applying such knowledge to improve coral health.

Ecological approaches to environmental assessment have traditionally focused on mortality as a metric to assess coral health, such as coral cover reductions, percentage of colony bleaching and/or mortality, and loss of individuals or species. Mortality is not an adequate metric of health or stress level, as it leaves little room for management or mitigation interventions. Additionally, ecological parameters can only provide correlation between putative stressors and effects, rather than actual causation. The application of molecular tools to assess sublethal stress levels in corals has increasingly gained attention and is being more broadly applied to identify the “smoking guns” in multi-stressor situations and provide metrics for evaluating the effectiveness of management interventions over periods of weeks to months, *versus* years to decades (Parkinson et al., 2020).

Molecular biomarkers, such as changes in protein expressions, enzymatic activity levels, gene expressions, and DNA damage, have been successfully developed and applied to capture sub-lethal stress levels in corals (e.g., Downs et al., 2005; Seneca et al., 2009; Kenkel et al., 2011; Richmond, 2011; Rougée, 2011; Edge et al., 2013; Seveso et al., 2013; Seneca and Palumbi, 2015; Murphy and Richmond, 2016). Since proteins directly affect organismal physiology and hence, represent functional adaptations (Feder and Walser, 2005; Tomanek, 2011), protein biomarkers are especially effective tools for assessing the sub-lethal stress responses in organisms. For example, following the ship grounding in Yap, Downs et al. (2006) conducted a field study to assess damage in corals using protein expressions in areas not visibly oiled and showed that elevated physiological stress, such as oxidative stress response and xenobiotic response, along with the presence of Benzo(a)pyrene-7,8-dihydrodiol-9,10-epoxide (BPDE) adducted proteins, provided the evidence for oil exposure, relative to corals from a reference site.

Working with corals, which are non-model organisms, is challenging and the field of coral molecular biomarker analysis (ecotoxicology) is still at a relatively early stage. No coral-specific antibodies are available commercially to analyze their protein expressions. However, previous studies suggest many key biomarker proteins may be highly conserved across metazoans (e.g., Barshis et al., 2010; Seveso et al., 2016), and therefore, in search of usable antibodies, commercially available antibodies (mostly made from vertebrates) were explored for application in corals by aligning protein sequences translated from the available coral transcriptomes, with the antibody sequences, selecting potentially compatible antibodies, and testing with extracted coral proteins. The initial protein studies, including the one presented here, were performed using enzyme-linked immunosorbent assays (ELISA) and Western blotting (e.g., Downs et al., 2006; 2012), targeting single protein per assay based on the existing knowledge. As technology became more available and affordable, the omics approach opened the door to investigating cellular responses without *a priori* hypotheses, which has been especially beneficial for non-model organisms for which we have limited knowledge about cellular and molecular responses/pathways (Parkinson et al., 2020). The more traditional protein assays, such as ELISA and Western blotting, are still useful in screening initial response time, and in identifying response patterns and dosages, since little information is often available to accurately predict organismal response direction and timing when working with non-model organisms like corals.

Maunalua Bay in Oahu, Hawaii is an important area for marine recreation and ocean activities, which receives discharges from nine sub-watersheds. The historical changes both on land and in the coastal ocean have been documented (Wolanski et al., 2009): Notably, poor land-use practices have resulted in the bay receiving large quantities of terrigenous sediment and a variety of pollutants. The channelization and concrete lining of streams, and other human activities within the adjacent watersheds have caused extensive habitat degradation and loss, which has resulted in the loss of coastal resources, including fishes and corals (Wolanski et al., 2009; Storlazzi et al., 2010; Presto et al., 2012). A range of stakeholders including local businesses, fishers, homeowners, canoe paddlers, divers, and resource managers joined together to address coral reef health declines and requested support from the research community in determining options for mitigation and recovery, which motivated us and others to assess the state of coral health along with water quality in Maunalua Bay. The urbanization and coastal development in the bay have created steep environmental gradients of anthropogenic stressors from the nearshore towards offshore in the bay, which ironically provided an opportunity for examining the impacts of urbanization on coral reefs.

In this study of corals in Maunalua Bay, we assessed protein expressions of stress response-related proteins in the lobe coral, *Porites lobata*, collected along the existing environmental gradient (e.g., Presto et al., 2012) from the mouth of the bay towards offshore. Our study aimed to 1) determine differences in sub-lethal cellular stress levels that corals experienced across different environments, 2) reveal the potential causes underlying such molecular stress responses, and 3) identify molecular markers that can be used for management purposes. We also tested sediment and water quality from the inshore areas from the bay for the presence of organic toxicants using gas chromatography-mass spectrometry (GC-MS). Our results revealed key stressors of concern and candidate coral biomarkers for anthropogenic stress levels using the widely distributed, pan-Pacific coral species, *P. lobata*.

## 2 Materials and methods

### 2.1 Coral biomarker analysis

#### 2.1.1 Coral collection

**FIGURE 1:**
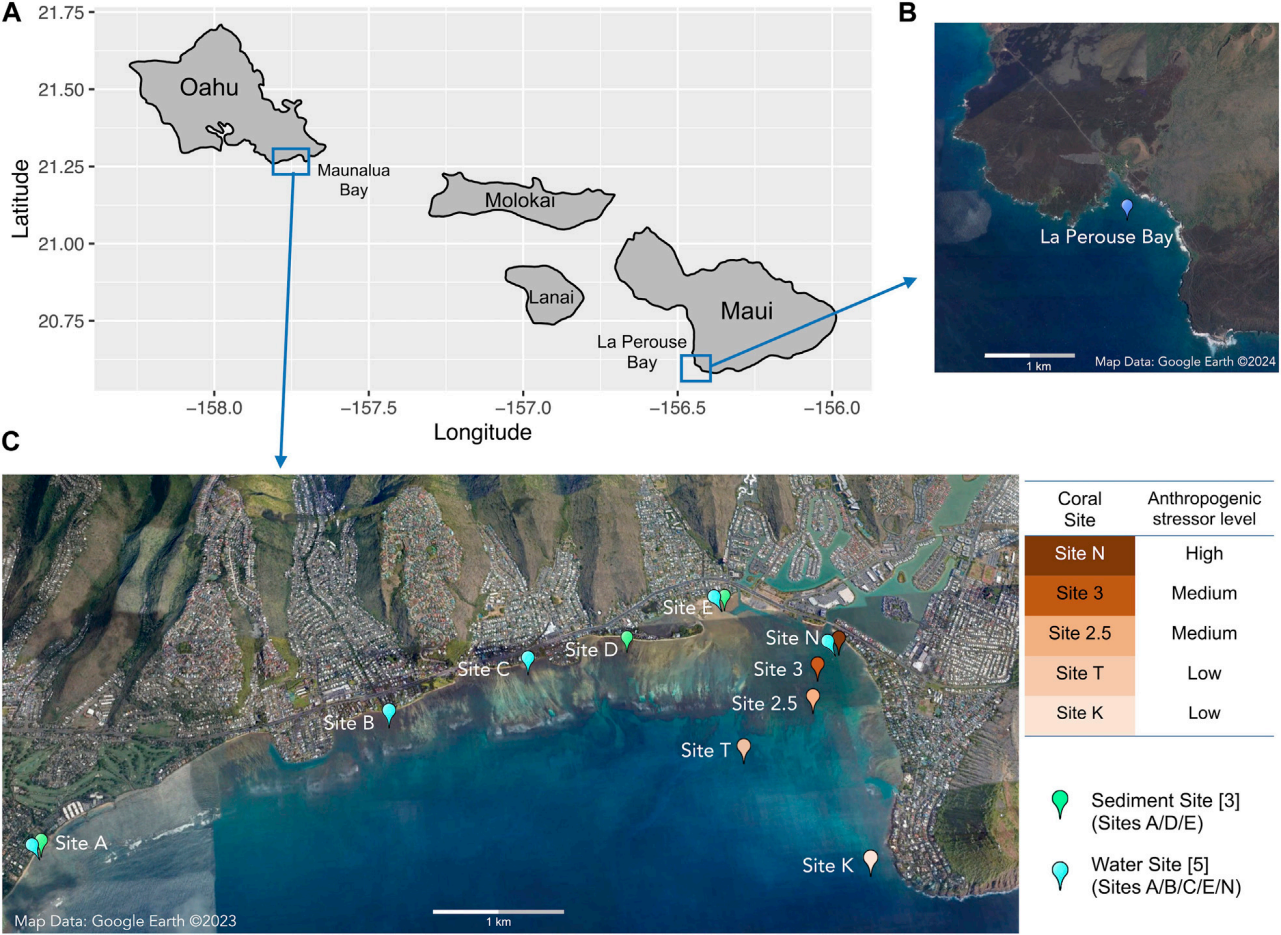
Map of **(A)** the Hawaiian Islands with sampling locations, **(B)** the reference site of Le Perouse Bay, Maui, and **(C)** the study site of Maunalua Bay, Oahu with sampling locations. Coral sampling sites are colored based on the anthropogenic stressor levels (water quality).

*Porites lobata* samples were collected from five locations along the existing environmental gradients in Maunalua Bay (Figure 1). We also selected a site away from coastal development and anthropogenic stressors as a reference site and sampled *P. lobata* from La Perouse Bay in Maui. At each site, 5–7 colonies of similar sizes with no apparent mortality or lesions were randomly selected, and small plugs (1 cm^2^) were obtained using a metal coral punch and a hammer, and flash frozen immediately by placing them in liquid nitrogen. All locations were relatively shallow (2–6 m), and environmental conditions were well characterized from previous studies, with inshore sites experiencing higher turbidity and fluctuations of temperature and salinity (Wolanski et al., 2009; Storlazzi et al., 2010; Presto et al., 2012; Tisthammer et al., 2021). All frozen corals were transported to the Kewalo Marine Laboratory and kept in a—80°C freezer until processed.

#### 2.1.2 Coral sample processing and ELISA

Samples were assayed following the methods from Downs (2005) and Downs et al. (2012). Briefly, the *P. lobata* nubbins were ground while frozen to a fine powder using liquid nitrogen, placed in microcentrifuge tubes along with 1,400 mL of a denaturing buffer (2% SDS, 50 mM Tris–HCl (pH 7.8), 15 mM dithiothreitol, 10 mM EDTA, 3% polyvinylpolypyrrolidone (wt/vol), 0.005 mM salicylic acid, 0.001% (v/v) dimethyl sulfoxide, 0.01 mM 4-(2-aminoethyl) benzenesulfonyl fluoride hydrochloride (AEBSF), 0.04 mM bestatin, 0.001 E−64, 2 mM phenylmethylsulfonyl fluoride, 2 mM benzamide, 5 mM a-amino-caproic acid, and 1 µg/100 mL pepstatin A). Samples were vortexed, heated at 93°C for 6 min, incubated at 25°C for 10 min, and centrifuged (13,500 g for 10 min). The middlephase supernatant was placed into a new tube, and protein concentrations were determined using the method of Ghosh et al. (1988).

We used the following 10 EnVirtue Biotechnologies, Inc. antibodies (generated in rabbits) that were specific to only the cnidarian animal targets, and not the coral’s dinoflagellate symbiont: 1) anti-ubiquitin (AB-U100), 2) anti-cnidarian heat-shock protein 70 (Hsp70) (AB-H101-CDN), 3) anti-cnidarian heat-shock protein 60 (Hsp60) (AB-H100-IN), 4) anti-cnidarian glutathione-S-transferase (GST) (AB-G100-MU), 5) anti-cnidarian copper/zinc superoxide dismutase (SOD) (cytosolic isoform, lot 1517), 6) anti-cnidarian glutathione peroxidase (GPx) (AB-GPX-IN), 7) anti-MutY (lot 3217), 8) anti-multi-xenobiotic resistance protein (MXR) (ABC family of proteins; P-glycoprotein 140 and 160; AB-MDR160), 9) anti-cnidarian protoporphyrinogen oxidase IX (lot 1945), and 10) anti-cnidarian heme oxygenase I (lot 3114). All antibodies were mono-specific polyclonal antibodies made against a synthetic 8–12 amino-acid residue polypeptide that reflects a specific and unique region of the target protein, which included sequences of cnidarian (*Nematostella vectenesis*, *Orbicella annularis*, *Acropora millepora*, and *Porites astreoides*). One-dimensional SDS-PAGE and Western blotting were performed to optimize the separation of target proteins and validate the use of specific antibodies for *P. lobata* protein extracts following the methods of Downs (2005) and Downs et al. (2012). The sequences of the antibodies and the images of blotting of these antibodies used on coral samples are available in Downs and Downs (2007), Figures 1–4) and Downs et al. (2012), Figure 2 and Electronic Supplementary Material). Antibodies and samples were optimized and the level of precision for each ELISA was determined using an 8 9 6 9 4 factorial design (Crowther, 2000). A Beckman-Coulter Biomek 2000 Laboratory Automation Workstation using 384-well microplates was used to conduct the ELISA assays. All samples were assayed in triplicate, and an eight-point calibrant curve using a calibrant relevant to each antibody was plated in triplicate for each plate.

**FIGURE 2:**
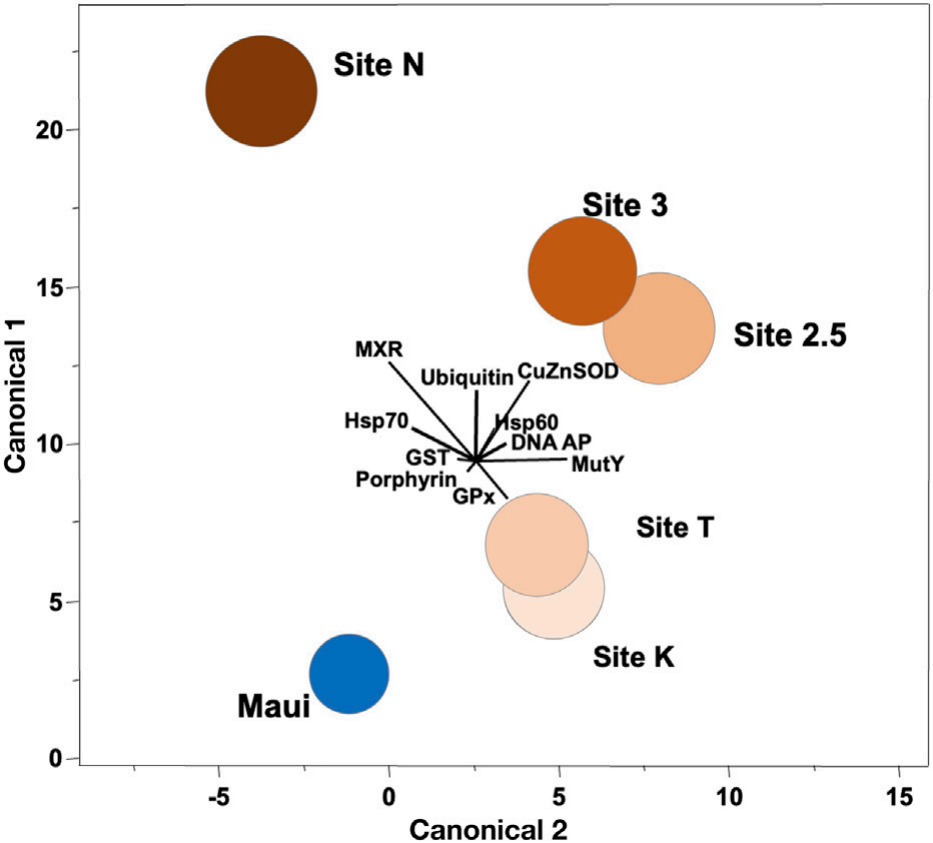
Canonical correlation of ten biomarkers in corals collected along the environmental gradient in Maunalua Bay. Colors of the sites correspond to those of Figure 1. Spheroids show the 95% confidence intervals around the distribution centroid of each site, and biplots show directions of original biomarker responses in canonical space.

#### 2.1.3 DNA damage analysis

DNA abasic or apurinic/apyrimidinic lesions (DNA AP sites) were used as an indicator of genetic damage. DNA was isolated from coral tissues according to the manufacturer’s instructions using the Dojindo pureDNA kit-Cell, Tissue (GK03-20; Dojindo Molecular Technologies) with one slight modification to address Maillard chemistry artifact. Coral tissues were added to the kit’s lysis buffer containing 100 mg of polyvinylpolypyrrolidone (PVPP, Sigma-Aldrich Corporation, St. Louis, Missouri, United States). DNA purity was determined spectrophotometrically using the 260/280 nm method (Sambrook and Russel, 2001). The DNA concentration was measured using an Invitrogen/Molecular Probes Quant-iT™ DNA Assay Kit, Broad Range (catalog # Q33130; Life Technologies Corporation) using a Bio-Tek FL800 fluorescent microplate reader (BioTeck Industries, Incorporated, Winooski, Vermont, United States). DNA AP (abasic site) sites were quantified using the Dojindo DNA Damage Quantification Kit-AP Site Counting (catalog # DK-02-10; Dojindo Molecular Technologies, Inc.) and conducted according to the manufacturer’s instructions.

#### 2.1.4 Data analysis

The results from ELISA and DNA AP sites were tested for normality using the Kolmogorov–Smirnov test (with Lilliefors’ correction) and for equal variance using the Levene Median test. When the data were normally distributed and homogeneous, a one-way analysis of variance (ANOVA) was employed. When data did not meet the homogeneity of variances requirement for one-way ANOVA, a Kruskal-Wallis One-Way Analysis of Variance on Ranks (Kruskal-Wallis H test) was used. When significant differences were found among treatment means, we used Tukey–Kramer Honestly Significant Difference (Tukey-HSD) test for the markers that met the parametric test assumptions and Dunn’s *post hoc* test for the rest, and applied the Holm-Sidak method as an exact alpha-level test to determine differences between pairwise populations (Holm, 1979; Sokal and Rohlf, 1995). Statistical significance was defined as *p*-value < 0.05. Correlation analysis was conducted using the distance from the mouth of the bay (21°16.878′, −157°42.688′) and protein expression levels using Pearson’s or Spearman’s correlation test. We used canonical correlation analysis (CCA) as a heuristic tool to illustrate how biomarkers could be used to discriminate among populations/sites. CCA reveals the basic relationships between two matrices, in our case those of the six sites (Sites N, 3, 2.5, K, T and Maui as a categorical location variable) and the biomarker data (total of 10 biomarkers, excluding heme oxygenase as no data for the reference site were collected). The CCA provided an objective statistical tool for determining if corals from the studied sites were significantly different from one another using sets of cellular biomarkers that are indicative of a cellular process and if so, which biomarkers contributed to those differences. This analysis required combining data from all six populations into one matrix, which we did by expressing biomarker responses in a given population as a proportion of their mean levels.

### 2.2 Sediment chemistry analysis (3 sites)

#### 2.2.1 Sample collection

Sediments were sampled at three locations, a) Waialae Beach Park (Site A), b) Paiko coastline (Site D), and c) Kuliouou Beach Park (Site E) in Maunalua Bay, in triplicate, except for Site E, which had four samples (Figure 1). At Site A, all samples were taken about 50 yards perpendicular from the shore between the two discharge points. At Site D, all three samples were taken about 4.5∼6 m from the shore at the passage located between residential houses to the beach. At Site E, three samples were taken just off the bottom substrate line at the low tide, and one sample was taken about 1.5 m offshore.

#### 2.2.2 Reagents

Dichloromethane and acetone were pesticide grade solvents (Fisher Scientific). Analytical standards were purchased from Ultra Scientific, RI, United States. Two stock standards were prepared. The first was SVOC stock which included: semi-volatile mixture (SVM-525) and organochlorine pesticide mixture (PPM-525E) (see table for list of analytes). The second was NP stock which included: nitrogen/phosphorous pesticide mixture 1 (NPM-525C) and nitrogen/phosphorous pesticide mixture 2 (NPM-525B). The internal standard solution (ISM- 510) was used for GC-MS quantitation. Sodium sulfate (Fisher Scientific) was baked at 200°C overnight and then pre-rinsed with hexane before use.

#### 2.2.3 Sample preparation

Marine sediment samples were placed in 125 mL pre-cleaned amber glass jars with Teflon-lined lids (I-Chem 300 series, VWR). Samples were collected, transported, and stored in the walk-in refrigerator until extraction. Sediment samples were extracted using an ASE 200 (Dionex Corporation, Sunnyvale, CA, United States). Approximately 5 g wet weight marine sediment was placed in a hexane rinsed aluminum dish and diatomaceous earth (Dionex Corporation, Sunnyvale, CA, United States) was added and ground until dry. This mixture was added to a 22 mL ASE extraction cell. Blank and spike (5 g/L = 5 ppm) samples were prepared in Ottawa Sand (mesh 200–300, Fisher Scientific) and diatomaceous earth. ASE extraction solvent was dichloromethane:acetone (1:1) run at 1,500 psi, 100°C for 5 min with flush of 50% ASE cell volume and nitrogen purge for 80 s. The ASE extract was passed through sodium sulfate, dried, and quantitatively transferred to a GC vial. Internal standards were added (acenaphthene-d10, phenanthrene-d10, and chrysene-d12) and the extract was analyzed by GC-MS.

#### 2.2.4 Instrument analysis

Two separate GC-MS methods were developed which included a semi-volatile/organochlorine (SVOC) method and a nitrogen/phosphorous (NP) method. These methods were the same except for the analyte list. Samples were run on a Varian 3800 GC/Saturn 2200 ion trap mass spectrometer (Varian Inc. Walnut Creek, CA, United States). The gas chromatograph was equipped with a 30 m VF-5ms column (0.25 mm i.d., 0.25 mm film) run at 1.1 mL/min helium with a pressure pulse of 45 psi for 0.8 min. The oven temperature started at 70°C with a 1-min hold, then was raised to 300°C at 4°C/min and held at 300°C for 2 min. A 1 uL sample was injected split/splitless at 250°C using a Varian CP-8400 autosampler. The transfer line temperature was 270°C, trap at 200°C, and manifold at 80°C. The mass spectrometer was run in full scan mode. Data was analyzed using Saturn GC-MS Workstation version 6.42.

#### 2.2.5 Quality control

A five-point standard curve was run for all compounds (0.01–10 ug/mL) which had a correlation coefficient greater than 0.99 with less than 15% standard deviation. Samples, blanks, and spikes were all run in duplicate reporting results as an average.

### 2.3 Water quality analysis (5 sites)

#### 2.3.1 Sample collection

Water samples were collected from five inshore sites in Maunalua Bay (Figure 1, Sites A/B/C/E/N). The samples were brought back to the Kewalo Marine Laboratory, and processed or used immediately for solid-phase extraction (3.2).

#### 2.3.2 Instrument analysis

Collected samples in solid phase cartridges were sent to Jupiter Environmental Laboratories (Jupiter, FL) for select organic compound analysis by GC-MS and LC-MS. Briefly, the cartridges were eluted with 2.5 mL of methanol twice to a final volume of 5 mL. The samples were allowed to settle and spun at 5 K for 4 min. The top layer was drawn off to avoid any salts that settled out of the cartridge. An open scan GC-MS analysis was performed and all peaks were checked against the NIST library. The open scan runs displayed few peaks, and when the Library search yielded nothing, high-performance liquid chromatography-tandem mass spectrometry (HPLC-MS/MS) was performed as an orthogonal discovery technology with much greater sensitivity. The samples were run against Multiple Reaction Monitoring transitions for triazine herbicides, chlorophenoxy herbicides, organophosphate pesticides, and pharmaceuticals and personal care products (PPCPs). Samples were listed as qualitatively positive if they were found to be above our LOQ, had a matching secondary ion and matching retention time when run by HPLC-MS/MS.

## 3 Results

### 3.1 Coral stress responses segregate over distance from the discharge

We conducted ordination analysis to assess if the aggregate expression patterns of biomarkers in *P. lobata* differed between sites. The analysis results showed clear segregation of the responses over distance from the mouth of the bay (Site N to Site K/T) along the canonical axis 1 (Figure 2). The inshore site (Site N) was characterized by signs of toxicant exposure, reflected in MXR, GST, and ubiquitin, and the mid-station sites (Site 3/2.5) had notable signs of oxidative damage reflected in SOD. The outer sites (Site K/T) with the lowest anthropogenic stressor level, also displayed some toxicant exposure signatures but were different from the rest of the sites. The reference site (Maui) was distinctly segregated from all sites, and was closest to the outer sites, suggesting the ordination results reflected well the water quality of the habitats where corals were sampled. Interestingly, toxicant exposure signatures of corals from the outer sites included signs of DNA damage reflected in DNA AP sites and MutY biomarkers, which may be due to pesticide exposure (from terrestrial runoff) or from chlorine or bromine exposure from swimming pool water discharges into that site from the nearby residential area (Portlock, east of Site K) (Richardson et al., 2010). When these pools are drained, the water is often discharged into the storm drains which empty into the ocean at the affected site.

**FIGURE 3:**
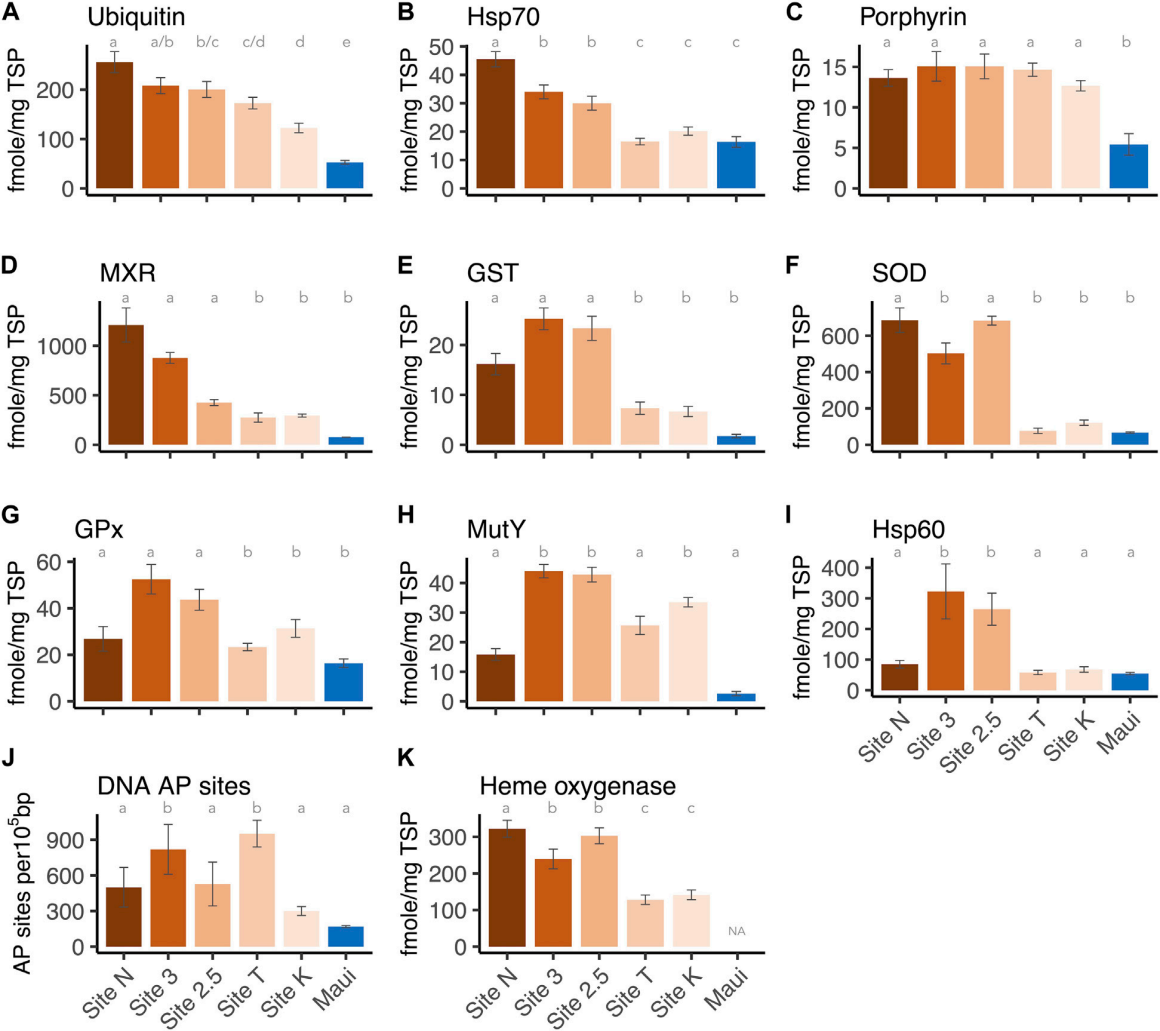
Expression levels of each biomarker per site: **(A)** Ubiquitin, **(B)** Hsp70, **(C)** Protoporphyrinogen oxidase IX, **(D)** MXR, **(E)** GST, **(F)** Cu/Zn SOD, **(G)** GPx, **(H)** Hsp60, **(J)** DNA AP sites, and **(K)** Heme oxygenase I. The letters above the bars indicate the statistically significant difference (Tukey-HSD or Dunn’s test with adjusted *p*-value < 0.05).

Each biomarker was also analyzed separately to determine how expression levels differed between sites. All markers showed overall differences among sites (one-way ANOVA or Kruskal-Wallis H test, *p*-values < 0.001, Supplementary Material S1). Post-hoc pairwise analysis revealed that Hsp 70 and ubiquitin showed the most variable expression levels between sites: Ubiquitin concentrations from all five sites in the bay were significantly higher than those from the Maui reference site (Tukey-HSD, adjusted *p*-values < 0.05), as well as the inshore site (Site N) having a significantly higher level than the outer and middle sites (Site K/T, 2.5) (Tukey-HSD, adjusted *p*-values < 0.05) (Figure 3A). The two middle sites (Site 3/2.5) also had significantly higher ubiquitin levels than the outer Site K (Tukey-HSD, adjusted *p*-values < 0.05), indicating ubiquitin as a sensitive biomarker of anthropogenic stressors/toxicant exposure. For Hsp70, on the other hand, the corals at the inshore and middle sites (Site N, 3/2.5) had significantly higher concentrations than those from Maui and the outer sites (Site K/T) (Tukey-HSD, adjusted *p*-value < 0.05), but the concentrations of the proteins from the corals at the outer sites did not differ from those of Maui (Figure 3B). This suggests that ubiquitin and Hsp70 are potentially useful biomarkers of anthropogenic stressors, but ubiquitin is more sensitive than Hsp70. Porphyrin concentrations in corals from all five sites in the bay were significantly higher than in those from Maui (Tukey-HSD adjusted *p*-value < 0.05), but within the bay, there were no differences (Figure 3C), suggesting ubiquitous organochlorine exposure and heightened antioxidant activity in corals within the bay. For MXR and GST, corals from the inshore and middle sites (Site N, 3/2.5) had significantly higher expression levels than those from Maui (Dunn’s test, adjusted *p*-value < 0.05), but not the offshore corals (Site K/T) ((Figures 3D, E), as seen in Hsp70. SOD showed a similar pattern, with the inner and middle site corals having significantly higher levels than Maui corals (Dunn’s test, adjusted *p*-value < 0.05), but corals from Site 3 did not differ from those in Maui (Figure 3F). GPx and MutY showed significantly higher levels only in the middle and offshore sites (Site 3/2.5, K) compared to Maui (Tukey-HSD/Dunn’s test, adjusted *p*-value < 0.05) (Figures 3G, H). Hsp60 was only higher in the middle site corals (Site 3/2.5) compared to those from Maui (Dunn’s test, adjusted *p*-value < 0.05) (Figure 3I), and DNA AP sites were significantly higher in Site 3 and Site T compared to Maui (Tukey-HSD, adjusted *p*-value < 0.05) (Figure 3A). Heme oxygenase, which was only tested among corals from the bay (no Maui), showed the inner and middle site corals (Site N, 3/2.5) with significantly higher expression levels than the outer site corals (Site K/T) (Tukey-HSD, adjusted *p*-value < 0.05), again reflecting the higher stress levels experienced by the inner and middles site corals.

### 3.2 Some biomarkers showed the dose-response relationship along the environmental gradient

**FIGURE 4:**
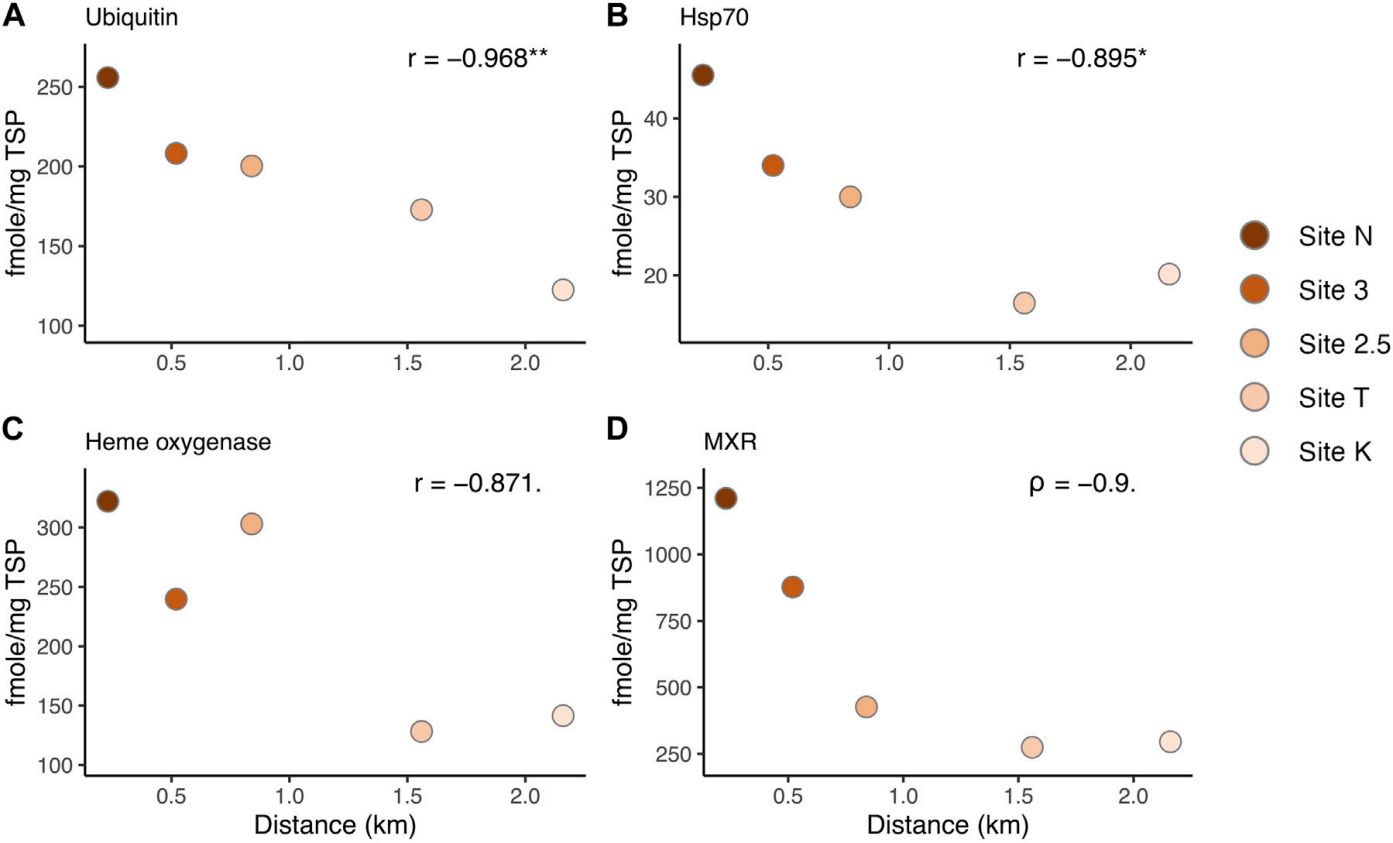
Correlation between protein biomarker expression levels and the distance from the mouth of the bay, representing the environmental (anthropogenic stressor) gradient: **(A)** Ubiquitin, **(B)** Hsp70, **(C)** Heme oxygenase I, and **(D)** MXR. r: Pearson’s correlation coefficient. Asterisks denote statistical significance (* = *p* < 0.05, ** = *p* < 0.01). Dots denote a marginally significant *p*-value (< 0.1).

Several of the biomarkers showed decreased responses in a linear fashion from inshore to offshore along the environmental gradient (Figure 4). Hsp70 and ubiquitin showed a significant correlation between the distances from the mouth of the bay (i.e., anthropogenic stressor levels) and the expression levels (Pearson test, ubiquitin: *p*-value = 0.007, Hsp70: *p*-value = 0.04, Figures 4A, B). MXR and heme oxygenase showed a marginally significant relationship (heme oxygenase: Pearson test, *p*-value = 0.055, MXR: Spearman test, *p*-value = 0.08). These results help direct the choice of biomarkers for future work depending on the stressors of interest. However, there was no breakpoint along the gradient in Maunalua Bay, where protein biomarker levels were as low as those found in corals from the reference site (Maui) (Figures 3, 4), suggesting the corals of this Bay are all under the influence of land-based sources of stress and pollution. Nonlinear responses were also observed in several biomarkers, including SOD and MutY. Studies have found that nonlinear responses do occur in corals (e.g., Gil, 2013; Bove et al., 2019), with some proteins and associated metabolic pathways responding differently based on exposure concentrations and timing.

### 3.3 Organic contaminants were present in inshore sediments and water

Various organic contaminants were found through our analysis of marine sediments collected from three discharge sites across Maunalua Bay (Table 1). At both Sites A and E, benzo [a]pyrene, benzo [b]fluoranthene, and benzo [k]fluoranthene were detected, indicating relatively ubiquitous presence of polycyclic aromatic hydrocarbons (PAHs) in inshore areas of the bay. At Site A, additional eight contaminants were found along with the suspected presence of chrysene and DDD/DDE. At Site E, one additional PAH (pyrene) was identified, while none were detected at Site D. Variations in detected contaminants among sites likely represent the sediment flushing/retention rates at particular sites and/or limitations or difficulties of detecting trace compounds from a single sampling method.

**TABLE 1:** Contaminants detected from sediment samples collected from inshore three sites in Maunalua Bay. We considered “Hit” (◎) as above 80% match with the NIST library, “Presence” (○) as below 80% match with NIST library, and ・represents suspected presence. Those with no symbols represent “not detected”.

Type	Contaminant	Site A	Site D	Site E
PAHs	benzo [a]pyrene	◎		○
PAHs	benzo [b]fluoranthene	◎		○
PAHs	benzo [k]fluoranthene	◎		○
PAHs	pyrene			◎
PAHs	benzo [a]anthracene	◎		
PAHs	phenanthrene	◎		
PAHs	anthracene	◎		
Pesticide	alpha-Chlordane	◎		
Pesticide	trans-Nonachlor	◎		
PAHs	benzo [ghi]perylene	○		
PAHs	ideno [1,2,3cd]pyrene	○		
PAHs	chrysene	・		
Pesticide	DDD/DDE	・		

Water chemistry was also analyzed from samples collected from five inshore sites across Maunalua Bay, which showed trace levels (<5 μg/L) of Bisphenol A (BPA) and oxybenzone in all samples (Table 2). Ultra trace levels of atrazine and simazine (<0.05 μg/L) were also found at Sites C and N. Relatively high concentration of herbicide dicamba (96.5 μg/L) was detected at Site N. Triazine herbicides were negative for all samples. Again, we do not know if the differences among sites for the ultra-trace level contaminants are due to the detection limit, rather than due to true water quality differences. However, the ubiquitous presence of BPA and oxybenzone in the nearshore waters is indisputable.

**TABLE 2:** Contaminants detected from water samples collected from five inshore sites in Maunalua Bay. Here, “Hit” (◎) is a compound concentration detected at a trace level (<5 μg/L), and “Presence” (○) is at an ultra-trace level (<0.05 μg/L). The symbol ◉ indicates a high Hit (96.5 μg/L). Those with no symbols represent “not detected”.

	Site A	Site B	Site C	Site E	Site N
BPA	◎	◎	◎	◎	◎
oxybenzone	◎	◎	◎	◎	◎
atrazene			○		○
simazine			○		○
dicamba					◉

## 4 Discussion

The documented declines in coral reef health are the result of multiple stressors acting in concert. Two specific requests from coral reef resource managers and stakeholders were 1) for techniques that could identify cause-and-effect relationships between putative stressors and coral health at the sublethal level, when interventions had the greatest potential for leading to positive outcomes and 2) for tools and associated metrics that would allow for determining the efficacy of management actions in real time, meaning over weeks to months rather than years to decades. The biomarker approach described here, which was modeled after those used for diagnosing and treating human health issues and maladies, met both needs. The elevated expression levels of stress induced proteins in corals from Maunalua Bay, especially those from inshore areas near discharge sites, relative to those from the reference site in Maui demonstrated that the sublethal stress in corals due to anthropogenic stressors (water quality) can be reliably detected. We also showed that some proteins, such as ubiquitin, Hsp70, and MXR, can be used to create a dose-response curve, which will help create the "thresholds’ for assessing the effects of individual environmental stressors, and those acting in concert. Proteomics/transcriptomics can show the stress response or adaptive mechanisms of corals to environmental stressors (e.g., Thomas et al., 2019; Tisthammer et al., 2021). However, to establish the stress threshold efficiently and cost-effectively, here we showed that a dozen of (or fewer) proteins can be used as diagnostic biomarkers.

The fact that the same proteins used to diagnose human ailments worked in corals is not surprising, as such responses occur at the cellular level, regardless of the “host” organism. In this study, antibodies raised against Cnidarians were used, but we have found many commercially available antibodies raised against vertebrates, such as catalase, cytochrome P450 A1, and Hsp90, to be effective in assessing protein expression levels in corals (unpublished data). This further affirms that practical biomarkers for diagnosing coral health can be available without extensive further studies. Indeed, deployable diagnosing tools in corals are gaining attention in recent years and are in development. For example, Meng et al. (2022) tested human urinalysis strips on corals and found that ketone and leukocytes were reliably detected by the strips.

Some of the proteins that were upregulated in corals exposed to reduced water quality, particularly toxicants, such as pesticides and polycyclic aromatic hydrocarbons (PAHs) ubiquitously detected in this study, are steroidal clearance/detoxification and regeneration enzymes that also play a role in reproduction (Rougée et al., 2006; Rougée et al., 2015; Rougée et al., 2021). Just as coral colony energetic allocation to maintenance and repair can compromise reproductive success including gamete quality and fecundity, so too can the allocation of key proteins to detoxification reduce the availability for reproduction and hence, population replenishment. As many coral reefs have suffered notable losses in coral colony density to levels below 10% (Eddy et al., 2021), populations are no longer capable of replenishment from local sources due to the Allee effect (Courchamp et al., 2008), where gamete dilution during spawning events prevents egg-sperm interactions, and hence, successful fertilization. Active coral reef restoration programs are viewed as essential for many reefs, particularly in the Atlantic/Caribbean, but will be unsustainable if transplanted corals are non-reproductive. Water quality is an essential parameter in the selection of sites for coral reef restoration, and the protein-based tools presented here can be used to assess the appropriateness of sites based on key biological responses, particularly on fecundity, gamete quality, and availability of essential steroidal proteins that can be allocated to reproduction on top of detoxification.

Since protein expression can be analyzed both qualitatively for identifying key stressors and quantitatively for determining threshold levels, protein expressions are a valuable tool for use by coral reef managers in efforts to protect and restore coral reefs. As the financial, institutional and human resources available for such tasks are limited, it is essential to use science-based approaches to develop management plans and to assess the efficacy of efforts to ensure positive outcomes. Our data demonstrate how the emerging tools and technologies can be applied. Future efforts to improve cellular diagnostics and advance the technologies for *in situ* analyses and monitoring will enhance the functionality and broader application of these tools.

## Data Availability

The datasets presented in this study can be found in online repositories. The names of the repository/repositories and accession number(s) can be found below: https://github.com/kahot/maunaluabay.
