# Supplementary material for: Differential molecular biomarker expression in corals over a gradient of water quality stressors in Maunalua Bay, Hawaii
Source: Front Physiol. 2024 Feb 21;15:1346045. doi: 10.3389/fphys.2024.1346045 (PMC10928694; doi:10.3389/fphys.2024.1346045)
Supplement: Supplementary file 1 [file DataSheet1.pdf]

Supplementary Material

Differential molecular biomarker expression in corals over a gradient of water quality stressors in Maunalua Bay, Hawaii

Kaho H. Tisthammer*, Jonathan A. Martinez, Craig A. Downs, Robert H. Richmond

*** Correspondence:** Corresponding Author: kahot@hawaii.edu

Supplementary Data

Statistical test results for each biomarker between the five sites.

1. Porphyrin

One Way Analysis of Variance

Normality Test: Passed (P > 0.050)

Equal Variance Test: Passed (P = 0.263)

Group Name	N	Missing	Mean	Std Dev	SEM
Maui reference	8	0	5.426	2.515	0.889
Site N	4	0	13.641	2.040	1.020
Site 3	4	0	15.052	3.644	1.822
Site 2.5	4	0	15.062	3.060	1.530
Site K	5	0	12.668	1.425	0.637
Site T	5	0	14.651	1.842	0.824

Source of Variation	DF	SS	MS	F	P
Between Groups	5	467.782	93.556	15.339	<0.001
Residual	24	146.380	6.099		
Total	29	614.162			

The differences in the mean values among the treatment groups are greater than would be expected by chance; there is a statistically significant difference (P = <0.001).

Power of performed test with alpha = 0.050: 1.000

All Pairwise Multiple Comparison Procedures (Holm-Sidak method):
Overall significance level = 0.05

Comparisons for factor:

Comparison	Diff of Means	t	Unadjusted P	Critical Level	Significant?
Site T vs. Maui reference	9.224	6.552	0.000	0.003	Y
Site 2.5 vs. Maui reference	9.635	6.371	0.000	0.004	Yes
Site 3 vs. Maui reference	9.626	6.365	0.000	0.004	Yes
Site N vs. Maui reference	8.215	5.432	0.000	0.004	Yes
Site K vs. Maui reference	7.242	5.143	0.000	0.005	Yes

Site 2.5 vs. Site K	2.394	1.445	0.161	0.005	No
Site 3 vs. Site K	2.385	1.439	0.163	0.006	No
Site T vs. Site K	1.983	1.269	0.216	0.006	No
Site 2.5 vs. Site N	1.421	0.814	0.424	0.007	No
Site 3 vs. Site N	1.411	0.808	0.427	0.009	No
Site T vs. Site N	1.010	0.609	0.548	0.010	No
Site N vs. Site K	0.973	0.587	0.562	0.013	No
Site 2.5 vs. Site T	0.411	0.248	0.806	0.017	No
Site 3 vs. Site T	0.402	0.242	0.810	0.025	No
Site 2.5 vs. Site 3	0.00941	0.00539	0.996	0.050	No

2. DNA AP sites

Kruskal-Wallis One Way Analysis of Variance on Ranks

Normality Test: Passed (P > 0.050)

Equal Variance Test: Failed (P = 0.015)

**Group**	**N**	**Missing**	**Median**	**25%**	**75%**
Maui reference	8	0	166.647	93.187	239.439
Site N	4	0	515.135	224.757	775.328
Site 3	4	0	760.060	483.665	1155.027
Site 2.5	4	0	418.477	300.090	755.138
Site K	5	0	310.975	255.620	338.978
Site T	6	0	942.875	732.670	1186.370

H = 20.489 with 5 degrees of freedom. (P = 0.001)

The differences in the median values among the treatment groups are greater than would be expected by chance; there is a statistically significant difference (P = 0.001)

To isolate the group or groups that differ from the others use a multiple comparison procedure.

Multiple Comparisons versus Control Group (Dunn's Method) :

**Comparison**	**Diff of Ranks**	**Q**	**P<0.05**
Site T vs Maui reference	19.708	4.014	Yes
Site 3 vs Maui reference	17.625	3.166	Yes
Site 2.5 vs Maui reference	11.625	2.088	No
Site N vs Maui reference	10.375	1.863	Do Not Test
Site K vs Maui reference	5.875	1.133	Do Not Test

Note: The multiple comparisons on ranks do not include an adjustment for ties.

3. Ubiquitin

One Way Analysis of Variance

Normality Test: Passed (P > 0.050)

Equal Variance Test: Passed (P = 0.698)

Group Name	N	Missing	Mean	Std Dev	SEM
Maui reference	8	0	52.875	20.392	7.210
Site N	6	0	255.833	52.331	21.364
Site 3	4	0	208.250	32.315	16.157
Site 2.5	6	0	200.333	39.662	16.192
Site K	6	0	122.500	23.586	9.629
Site T	6	0	172.833	28.951	11.819

Source of Variation	DF	SS	MS	F	P
Between Groups	5	175408.181	35081.636	30.440	<0.001
Residual	30	34574.125	1152.471		
Total	35	209982.306			

The differences in the mean values among the treatment groups are greater than would be expected by chance; there is a statistically significant difference (P = <0.001).

Power of performed test with alpha = 0.050: 1.000

All Pairwise Multiple Comparison Procedures (Holm-Sidak method):
Overall significance level = 0.05

Comparisons for factor:

Comparison	Diff of Means	t	Unadjusted P	Critical Level	Significant?
Site N vs. Maui reference	202.958	11.070	0.000	0.003	Yes
Site 2.5 vs. Maui reference	147.458	8.043	0.000	0.004	Yes
Site 3 vs. Maui reference	155.375	7.474	0.000	0.004	Yes
Site N vs. Site K	133.333	6.803	0.000	0.004	Yes
Site T vs. Maui reference	119.958	6.543	0.000	0.005	Yes
Site N vs. Site T	83.000	4.235	0.000	0.005	Yes
Site 2.5 vs. Site K	77.833	3.971	0.000	0.006	Yes
Site 3 vs. Site K	85.750	3.913	0.000	0.006	Yes
Site K vs. Maui reference	69.625	3.798	0.001	0.007	Yes
Site N vs. Site 2.5	55.500	2.832	0.008	0.009	Yes
Site T vs. Site K	50.333	2.568	0.015	0.010	No
Site N vs. Site 3	47.583	2.171	0.038	0.013	No
Site 3 vs. Site T	35.417	1.616	0.117	0.017	No
Site 2.5 vs. Site T	27.500	1.403	0.171	0.025	No
Site 3 vs. Site 2.5	7.917	0.361	0.720	0.050	No

4. Hsp70

One Way Analysis of Variance

Normality Test: Passed (P > 0.050)

Equal Variance Test: Passed (P = 0.634)

Group Name	N	Missing	Mean	Std Dev	SEM
Maui reference	8	0	16.375	5.041	1.782
Site N	6	0	45.500	6.595	2.693
Site 3	4	0	34.000	4.830	2.415
Site 2.5	6	0	30.000	6.033	2.463
Site K	6	0	20.167	3.545	1.447
Site T	6	0	16.500	2.881	1.176

**Source of Variation**	**DF**	**SS**	**MS**	**F**	**P**
Between Groups	5	4119.847	823.969	32.884	<0.001
Residual	30	751.708	25.057		
Total	35	4871.556			

The differences in the mean values among the treatment groups are greater than would be expected by chance; there is a statistically significant difference (P = <0.001).

Power of performed test with alpha = 0.050: 1.000

All Pairwise Multiple Comparison Procedures (Holm-Sidak method):
Overall significance level = 0.05

Comparisons for factor:

**Comparison**	**Diff of Means**	**t**	**Unadjusted P**	**Critical Level**	**Significa**
Site N vs. Maui reference	29.125	10.774	0.000	0.003	Yes
Site N vs. Site T	29.000	10.034	0.000	0.004	Yes
Site N vs. Site K	25.333	8.766	0.000	0.004	Yes
Site 3 vs. Maui reference	17.625	5.750	0.000	0.004	Yes
Site 3 vs. Site T	17.500	5.416	0.000	0.005	Yes
Site N vs. Site 2.5	15.500	5.363	0.000	0.005	Yes
Site 2.5 vs. Maui reference	13.625	5.040	0.000	0.006	Yes
Site 2.5 vs. Site T	13.500	4.671	0.000	0.006	Yes
Site 3 vs. Site K	13.833	4.281	0.000	0.007	Yes
Site N vs. Site 3	11.500	3.559	0.001	0.009	Yes
Site 2.5 vs. Site K	9.833	3.402	0.002	0.010	Yes
Site K vs. Maui reference	3.792	1.403	0.171	0.013	No
Site K vs. Site T	3.667	1.269	0.214	0.017	No
Site 3 vs. Site 2.5	4.000	1.238	0.225	0.025	No
Site T vs. Maui reference	0.125	0.0462	0.963	0.050	No

5. Hsp60

One Way Analysis of Variance

Normality Test: Failed (P = <0.001)

Test execution ended by user request, ANOVA on Ranks begun

Kruskal-Wallis One Way Analysis of Variance on Ranks

**Group**	**N**	**Missing**	**Median**	**25%**	**75%**
Maui reference	8	0	53.500	37.500	62.000
Site N	6	0	71.500	68.000	106.000
Site 3	4	0	258.500	200.000	445.000
Site 2.5	6	0	221.000	188.000	392.000
Site K	6	0	70.500	52.000	84.000
Site T	6	0	61.500	42.000	73.000

H = 23.380 with 5 degrees of freedom. (P = <0.001)

The differences in the median values among the treatment groups are greater than would be expected by chance; there is a statistically significant difference (P = <0.001)

To isolate the group or groups that differ from the others use a multiple comparison procedure.

Multiple Comparisons versus Control Group (Dunn's Method) :

Comparison	Diff of Ranks	Q	P<0.05
Site 3 vs Maui reference	22.188	3.439	Yes
Site 2.5 vs Maui reference	21.188	3.724	Yes
Site N vs Maui reference	8.688	1.527	No
Site K vs Maui reference	5.271	0.926	Do Not Test
Site T vs Maui reference	2.188	0.384	Do Not Test

Note: The multiple comparisons on ranks do not include an adjustment for ties.

6. MXR

One Way Analysis of Variance

Normality Test: Failed (P = <0.001)

Test execution ended by user request, ANOVA on Ranks begun

Kruskal-Wallis One Way Analysis of Variance on Ranks

Group	N	Missing	Median	25%	75%
Maui reference	8	0	75.500	63.500	87.500
Site N	6	0	1268.000	771.000	1630.000
Site 3	4	0	883.000	785.000	970.000
Site 2.5	6	0	437.000	356.000	487.000
Site K	6	0	302.500	281.000	327.000
Site T	6	0	255.500	186.000	331.000

H = 32.065 with 5 degrees of freedom. (P = <0.001)

The differences in the median values among the treatment groups are greater than would be expected by chance; there is a statistically significant difference (P = <0.001)

To isolate the group or groups that differ from the others use a multiple comparison procedure.

Multiple Comparisons versus Control Group (Dunn's Method) :

Comparison	Diff of Ranks	Q	P<0.05
Site N vs Maui reference	27.833	4.892	Yes
Site 3 vs Maui reference	25.750	3.991	Yes
Site 2.5 vs Maui reference	18.500	3.251	Yes
Site K vs Maui reference	10.333	1.816	No
Site T vs Maui reference	10.167	1.787	Do Not Test

Note: The multiple comparisons on ranks do not include an adjustment for ties.

7. GST

One Way Analysis of Variance

Normality Test: Failed (P = 0.008)

Test execution ended by user request, ANOVA on Ranks begun

Kruskal-Wallis One Way Analysis of Variance on Ranks

Group	N	Missing	Median	25%	75%
Maui reference	8	0	2.000	1.000	2.000
Site N	6	0	18.500	10.000	20.000
Site 3	4	0	24.500	22.000	28.500
Site 2.5	6	0	25.000	23.000	26.000
Site K	6	0	6.000	6.000	9.000
Site T	6	0	7.000	5.000	10.000

H = 30.928 with 5 degrees of freedom. (P = <0.001)

The differences in the median values among the treatment groups are greater than would be expected by chance; there is a statistically significant difference (P = <0.001)

To isolate the group or groups that differ from the others use a multiple comparison procedure.

Multiple Comparisons versus Control Group (Dunn's Method) :

Comparison	Diff of Ranks	Q	P<0.05
Site 3 vs Maui reference	26.938	4.175	Yes
Site 2.5 vs Maui reference	26.188	4.602	Yes
Site N vs Maui reference	18.688	3.284	Yes
Site T vs Maui reference	10.771	1.893	No
Site K vs Maui reference	10.021	1.761	Do Not Test

Note: The multiple comparisons on ranks do not include an adjustment for ties.

8. MutY

One Way Analysis of Variance

Normality Test: Failed (P = 0.015)

Test execution ended by user request, ANOVA on Ranks begun

Kruskal-Wallis One Way Analysis of Variance on Ranks

Group	N	Missing	Median	25%	75%
Maui reference	8	0	2.500	2.000	3.000
Site N	6	0	17.000	11.000	20.000
Site 3	4	0	43.500	41.000	47.000
Site 2.5	6	0	40.500	39.000	45.000
Site K	6	0	32.000	32.000	36.000
Site T	6	0	23.500	19.000	34.000

H = 31.669 with 5 degrees of freedom. (P = <0.001)

The differences in the median values among the treatment groups are greater than would be expected by chance; there is a statistically significant difference (P = <0.001)

To isolate the group or groups that differ from the others use a multiple comparison procedure.

Multiple Comparisons versus Control Group (Dunn's Method) :

**Comparison**	**Diff of Ranks**	**Q**	**P<0.05**
Site 3 vs Maui reference	27.500	4.262	Yes
Site 2.5 vs Maui reference	26.000	4.569	Yes
Site K vs Maui reference	18.250	3.207	Yes
Site T vs Maui reference	13.667	2.402	No
Site N vs Maui reference	7.750	1.362	Do Not Test

Note: The multiple comparisons on ranks do not include an adjustment for ties.

9. SOD

One Way Analysis of Variance

Normality Test: Failed (P = 0.027)

Test execution ended by user request, ANOVA on Ranks begun

Kruskal-Wallis One Way Analysis of Variance on Ranks

**Group**	**N**	**Missing**	**Median**	**25%**	**75%**
Maui reference	8	0	71.000	46.500	77.000
Site N	6	0	722.500	560.000	838.000
Site 3	4	0	511.000	408.500	596.500
Site 2.5	6	0	672.000	624.000	739.000
Site K	6	0	106.500	92.000	158.000
Site T	6	0	77.500	48.000	93.000

H = 29.302 with 5 degrees of freedom. (P = <0.001)

The differences in the median values among the treatment groups are greater than would be expected by chance; there is a statistically significant difference (P = <0.001)

To isolate the group or groups that differ from the others use a multiple comparison procedure.

Multiple Comparisons versus Control Group (Dunn's Method) :

**Comparison**	**Diff of Ranks**	**Q**	**P<0.05**
Site N vs Maui reference	22.604	3.973	Yes
Site 2.5 vs Maui reference	22.438	3.943	Yes
Site 3 vs Maui reference	16.188	2.509	No
Site K vs Maui reference	8.271	1.454	Do Not Test
Site T vs Maui reference	1.521	0.267	Do Not Test

Note: The multiple comparisons on ranks do not include an adjustment for ties.

10. GPx

One Way Analysis of Variance

Normality Test: Passed (P > 0.050)

Equal Variance Test: Passed (P = 0.298)

Group Name	N	Missing	Mean	Std Dev	SEM
Maui reference	8	0	16.375	5.041	1.782
Site N	6	0	26.833	12.968	5.294
Site 3	4	0	52.500	12.715	6.357
Site 2.5	6	0	43.667	11.057	4.514
Site K	6	0	31.333	9.416	3.844
Site T	6	0	23.333	3.933	1.606

Source of Variation	DF	SS	MS	F	P
Between Groups	5	4964.292	992.858	11.301	<0.001
Residual	30	2635.708	87.857		
Total	35	7600.000			

The differences in the mean values among the treatment groups are greater than would be expected by chance; there is a statistically significant difference (P = <0.001).

Power of performed test with alpha = 0.050: 1.000

Multiple Comparisons versus Control Group (Holm-Sidak method):
Overall significance level = 0.05

Comparisons for factor:

Comparison	Diff of Means	t	Unadjusted P	Critical Level	Significant?
Maui reference vs. Site 3	36.125	6.294	0.000	0.010	Yes
Maui reference vs. Site 2.5	27.292	5.391	0.000	0.013	Yes
Maui reference vs. Site K	14.958	2.955	0.006	0.017	Yes
Maui reference vs. Site N	10.458	2.066	0.048	0.025	No
Maui reference vs. Site T	6.958	1.375	0.179	0.050	No

11. Heme Oxygenase

One Way Analysis of Variance

Data source: Heme Oxygenase

Normality Test: Passed (P > 0.050)

Equal Variance Test: Passed (P = 0.556)

Group Name	N	Missing	Mean	Std Dev	SEM
Site N	6	0	322.000	57.131	23.324
Site 3	4	0	239.750	53.749	26.874
Site 2.5	6	0	303.000	53.153	21.699
Site K	6	0	141.500	32.617	13.316
Site T	6	0	128.167	31.720	12.950

Source of Variation	DF	SS	MS	F	P
Between Groups	4	191895.881	47973.970	22.308	<0.001
Residual	23	49463.083	2150.569		
Total	27	241358.964			

The differences in the mean values among the treatment groups are greater than would be expected by chance; there is a statistically significant difference (P = <0.001).

Power of performed test with alpha = 0.050: 1.000

All Pairwise Multiple Comparison Procedures (Holm-Sidak method):
Overall significance level = 0.05

Comparisons for factor:

Comparison	Diff of Means	t	Unadjusted P	Critical Level	Significant?
Site N vs. Site T	193.833	7.240	0.000	0.005	Yes
Site N vs. Site K	180.500	6.742	0.000	0.006	Yes
Site 2.5 vs. Site T	174.833	6.530	0.000	0.006	Yes
Site 2.5 vs. Site K	161.500	6.032	0.000	0.007	Yes
Site 3 vs. Site T	111.583	3.728	0.001	0.009	Yes
Site 3 vs. Site K	98.250	3.282	0.003	0.010	Yes
Site N vs. Site 3	82.250	2.748	0.011	0.013	Yes
Site 2.5 vs. Site 3	63.250	2.113	0.046	0.017	No
Site N vs. Site 2.5	19.000	0.710	0.485	0.025	No
Site K vs. Site T	13.333	0.498	0.623	0.050	No
